# Sarcopenic Obesity: An Emerging Public Health Problem

**DOI:** 10.14336/AD.2021.1006

**Published:** 2022-04-01

**Authors:** Tong Ji, Yun Li, Lina Ma

**Affiliations:** Department of Geriatrics, Xuanwu Hospital, Capital Medical University, National Research Center for Geriatric Medicine, Beijing 100053, China

**Keywords:** Sarcopenic obesity, older adults, pathogenesis, diagnostic criteria, prevalence, treatment

## Abstract

Population aging and the obesity epidemic are important global public health problems that pose an unprecedented threat to the physical and mental health of the elderly and health systems worldwide. Sarcopenic obesity (SO) is a new category of obesity and a high-risk geriatric syndrome in the elderly. SO is associated with many adverse health consequences such as frailty, falls, disability, and increased morbidity and mortality. The core mechanism of SO is the vicious circle between myocytes and adipocytes. In order to implement effective prevention and treatment strategies and reduce adverse clinical outcomes, it is essential to further our understanding of SO in the elderly. Herein, we reviewed the definition, diagnosis, epidemiology, pathogenesis, and treatment of SO in older adults.

With the acceleration of aging of world population, the proportion of aging population over 60 years old is going to 21% by 2050,and more than 5% of the population over 80 years old [1, 2]. In the background of aging population crisis, the prevalence of sarcopenic obesity (SO) is increasing. The incidence of SO in the elderly the world over is about 11%, gradually increased with age [3]. There is no consensus on the definition and diagnostic criteria of SO at present. SO is an emerging complex geriatric syndrome characterized by a dual burden of sarcopenia (low muscle mass, reduced muscle strength and physical dysfunction) and excess fat, resulting in many adverse clinical complications such as frailty, falls, disability, immobility, fractures, cardiometabolic and respiratory diseases, cancer, and increased mortality [4-9].

The pathophysiological mechanism of SO is rather complicated and indefinite (Fig. 1). The main etiological factors include age-related changes in body composition, sex-specific hormonal changes, chronic low-grade inflammation, insulin resistance, sedentary behavior, and unhealthy diet [5, 10]. Inflammation, oxidative stress, and insulin resistance are considered to be the key factors in the development of SO [5, 11, 12]. As the evidence accumulated, optimal diet and exercise strategies are of cardinal importance for preventing and treating SO and to combating the adverse outcomes. There are some potential and emerging treatments for SO, for instance, pharmacological interventions (testosterone supplementation, selective androgen receptor modulators, myostatin inhibitors, and anti-obesity drugs), electrical acupuncture and whole-body electromyostimulation, and A2B agonist [13-17].

In this article, we discuss and update the definition, diagnostic criteria, epidemiology, pathogenesis, and therapeutic approach of SO in the elderly.

**Figure 1.**
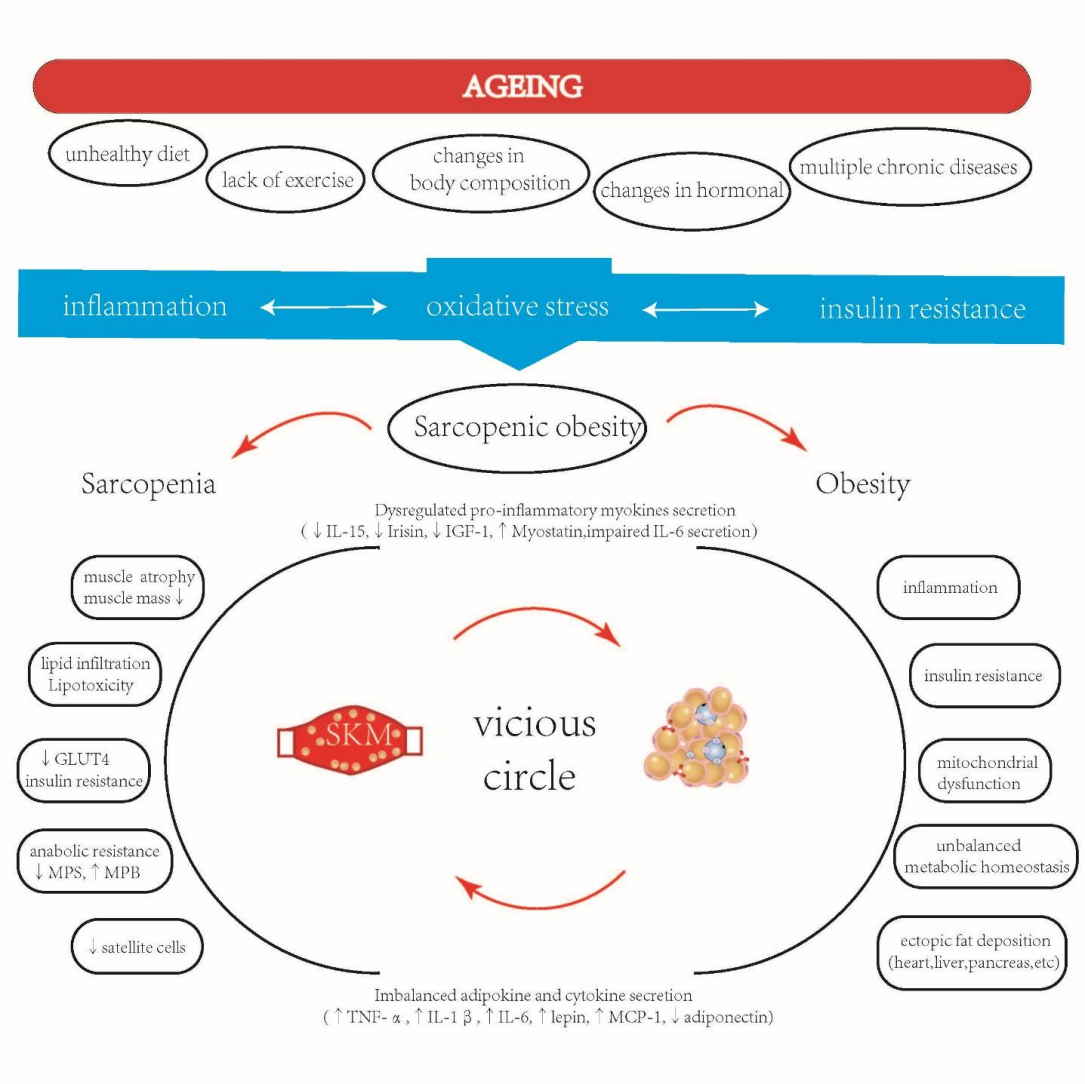
The potential etiology and pathogenesis of sarcopenic obesity (SO). In the process of aging, an unhealthy diet, sedentary habits, changes in body composition, hormone changes and a variety of chronic diseases in elderly cause chronic low-grade inflammation, oxidative stress, and insulin resistance, resulting in SO. The core mechanism of SO is the vicious circle between myocytes and adipocytes. SKM, skeletal muscle; MPS, skeletal muscle protein synthesis; MPB, muscle protein breakdown; GLUT4, glucose transporter type 4; IGF-1, insulin-like growth factor 1; IL, interleukin; MCP-1, monocyte chemoattractant protein 1.

## Definition and measurement

Baumgartner first proposed the concept of "Sarcopenic Obesity" in 2000 and defined it as a phenotype of co-presence of sarcopenia and obesity [18]. This definition was supported by a more recent critical appraisal of the definition and diagnostic criteria of SO based on a systematic review which noted that most existing studies defined SO based on the co-existence of obesity and sarcopenia [19] (Table 1). SO is a complex geriatric syndrome characterized by an aged-associated reduced muscle mass and dysfunction and excess adiposity [11, 20]. Therefore, individuals with SO have a double burden of malnutrition and are at an increased risk of frailty, disability, morbidity, and mortality. Due to the lack of consensus on the definition and diagnostic criteria for SO, accurate diagnostic assessment of SO is extremely challenging.

**Table 1:** Diagnostic criteria of sarcopenic obesity.

Author, year, and study name		Definition of Sarcopenia		Definition of obesity
Diagnostic criteria	Muscle Mass	Muscle Strength	Physical Performance	
Baumgartner, 2000 [18]	DXA:ASM/ht^2^ < 7.26kg/m^2^ (M) ASM/ht^2^ <5.45kg/m^2^ (F)	/	/	PBF>27%(M) PBF>38%(F)
Newman, 2003 [21]	DXA:ASM/ht^2^<7.23kg/m^2^(M) ASM/ht^2^<5.67kg/m^2^(F)	/	/	BMI≥30kg/m^2^
Baumgartner, 2004 [22] New Mexico Aging Process Study	DXA:ASM/ht^2^<7.26kg/m^2^(M) ASM/ht^2^<5.45kg/m^2^(F)	/	/	PBF>27%(M) PBF>38%(F)
Kim T.N,2009, The Korean sarcopenic obesity study [23]	DXA:ASM/ht^2^ < 7.26 kg/m^2^ (M) ASM/ht^2^< 5.45 kg/m^2^ (W)	/	/	PBF>27%(M) PBF>38%(F)
Cruz-Jentoft, 2010, EWGSOP [24]	DXA: ASM/ht^2^<7.26kg/m^2^(M) ASM/ht^2^<5.50kg/m^2^(F)(Rosetta Study) DXA:ASM/ht^2^<7.25kg/m^2^(M) ASM/ht^2^<5.67kg/m^2^(F)(health ABC study) DXA:ASM/ht^2^<7.23kg/m^2^(M) ASM/ht^2^<5.67kg/m^2^(F)(health ABC study) DXA: Residuals of linear regression on appendicular lean mass adjusted for fat mass as well as height:-2.29 (M), -1.73 (W) BIA:SM/ht^2^<8.87kg/m^2^ (W) SM/ht^2^<6.42kg/m^2^ (F) BIA: absolute muscle mass/ht^2^ severe<8.50kg/m^2^(W); <5.75kg/m^2^ (F) Moderate 8.51-10.75 kg/m^2^ (W); 5.76-6.75kg/m^2^ (F)	Handgrip<30 kg (M) Handgrip<20 kg (F) Handgrip based on BMI category: Men: BMI ≤24 ≤29kg BMI24.1-26 ≤30kg BMI26.1-28 ≤30kg BMI>28 ≤32kg Women: BMI ≤23 ≤17kg BMI23.1-26≤17.3kg BMI26.1-29≤18kg BMI >29 ≤21kg	GS<0.8 m/s (4 m) Or <1.0 m/s (6 m) SPPB≤8 points score	/
Fielding, 2011, IWGS [25]	DXA:ASM/ht^2^≤7.23 kg/m^2^(M) ASM/ht^2^≤5.67 kg/m^2^(F)	/	GS<1.0 m/s (6 m)	/
Studenski, 2014, FNIH [26]	DXA:ALM<19.75kg (W) ALM<15.02kg (F) DXA:ALM/BMI<0.789 (W) ALM/BMI<0.512 (F)	Handgrip <26 kg (M) Handgrip <16kg (F) Handgrip:BMI<1.0 (M) Handgrip:BMI<0.56 (F)	/	/
Chen LK, 2014, AWGS [27]	DXA:ASM/ht^2^<7.0kg/m^2^(M) ASM/ht^2^<5.4kg/m^2^(F) BIA:ASM/ht^2^<7.0kg/m^2^(M) ASM/ht^2^<5.7kg/m^2^(F)	Handgrip<26 kg (M) Handgrip<18kg (F)	GS<0.8 m/s (6 m)	/
Chuang 2015 [28]	DXA:TSM/ht^2^< 11.45 kg/m^2^(M) TSM//ht^2^<8.51 kg/m^2^(F)	/	/	WC ≥ 90cm (M) WC ≥ 80 cm (F)
Cruz-Jentoft, 2019, EWGSOP2 [29]	Use SARC-F questionnaire to find subjects with sarcopenia DXA/BIA:ASM<20kg(M) ASM<15kg(F) DXA/BIA:ASM/ht^2^<7.0kg/m^2^(M) ASM/ht^2^<6.0kg/m^2^(F)	Handgrip<27 kg (M) Handgrip<16kg (F) Chair stand>15s for five rises	GS≤0.8 m/s (6 m) SPPB≤8 point score TUG≥20s 400m walk test: non-cpmpletion or 6 min for completion	/
Chen LK, 2019, AWGS2 [30]	Use calf circumference, SARC-F or SARC-calf questionnaire to find subjects with sarcopenia DXA:ASM/ht^2^<7.0kg/m^2^(M) ASM/ht^2^<5.4kg/m^2^(F) BIA:ASM/ht^2^<7.0kg/m^2^(M) ASM/ht^2^<5.7kg/m^2^(F)	Handgrip<28 kg (M) Handgrip<18kg (F)	GS<1.0 m/s (6 m), or SPPB≤9 points score, or 5-time chair stand test ≥12 seconds	/

Notes: M, Male; F, Female; ASM, appendicular skeletal muscle mass; TSM, total skeletal muscle mass; ALM, appendicular lean muscle; GS, gait speed; MAMC, midarm muscle circumference; BMI, body mass index; PBF, percentage of body fat; WC, waist circumference; DXA, dual-energy X-ray absorptiometry; BIA, bioelectrical impedance analysis; EWGSOP, European Working Group on Sarcopenia in Older People; FNIH, Foundation for the National Institutes of Health; IWGSP, International Working Group on Sarcopenia; AWGS, Asian Working Group for Sarcopenia

Further, previous studies were characterized by significant differences in the measurement methods used to define sarcopenia and obesity (Table 2). A systematic review reported that there are 19 methods to evaluate sarcopenia and 10 methods to evaluate obesity. The most used methods to define sarcopenia and obesity are appendicular skeletal muscle (ASM) divided by weight (ASM/wt) or adjusted by height in meters squared (ASM/h^2^) and body mass index (BMI) or percentage of body fat (PBF), respectively [19]. Rough indicators such as weight and BMI are not recommended for the assessment of body composition in the elderly as these cannot distinguish between fat and muscle mass. Dual-energy X-ray absorptiometry (DXA) is a reliable technique for body composition analysis owing to its safety, repeatability, and accuracy; however, it is associated with a risk of radiation exposure [31]. Bioimpedance analysis (BIA) is a quick and portable technique for measuring body composition [32], which is suitable for large-scale epidemiological investigations and can replace DXA. Although computed tomography and magnetic resonance imaging are more accurate body composition analysis methods, they have limited clinical applications because of their high cost, radiation exposure, and need for qualified personnel [33].

**Table 2:** Different measurement methods of sarcopenic obesity.

Sarcopenia				Obesity
Muscle Mass	Muscle Strength	Physical Performance	Adiposity	Fat Mass
DXA(ASM/h^2^, ASM/wt, etc) Anthropometry (MAMC, calf circumference) BIA(ASM/h^2^, ASM/wt, etc) Ultrasonography, CT, MRI	HGS maximal knee extensor strength	GS TUG SPPB	Anthropometry (BMI, WC) DXA(PBF) BIA(PBF)	CT MRI

Abbreviations: DXA, dual-energy X-ray absorptiometry; ASM/wt, appendicular skeletal muscle divided by Weight; ASM/h^2^, appendicular skeletal muscle divided by height in meters squared; MAMC, mid-arm muscle circumference; BIA, bioelectrical impedance analysis; CT, computed tomography scan; MRI, magnetic resonance imaging; HGS,hand grip strength; GS, gait speed; TUG, timed up-and-go; SPPB, short physical performance battery; PBF, percentage of body fat.

## Criteria

### Definition of sarcopenia

Sarcopenia was first defined by Rosenberg in 1989 [34]. Sarcopenia refers to a group of age-related syndromes of decreased skeletal muscle content, decreased muscle strength, and muscle dysfunction, which can cause weakness, disability, and falls [35]. The ICD-10 code for sarcopenia was introduced in 2016 (M62.84), facilitating the assessment, diagnosis, and treatment of sarcopenia [36].

### Diagnosis of sarcopenia

Sarcopenia is defined by a variety of variables such as skeletal muscle mass (SMM), muscle strength, and physical performance (Table 2). SMM can be calculated using the following measurements: 1) ASM / ht^2^ [24]; 2) ASM/wt [37]; 3) based on residual height correction and total fat muscle mass [38]; 4) ASM adjusted by BMI [26]; 5) unadjusted or absolute appendicular lean muscle [26]; and 6) unadjusted or adjusted body mass, height, or BMI [24]. Studies have shown that SMM is not linearly related to muscle strength. Muscle strength decays faster than SMM and is a more valuable indicator of the overall health of the elderly [39]. Muscle strength can be evaluated by measuring grip strength using a hand dynamometer, or by measuring knee extension strength [40]. The assessment measures of physical performance include gait speed, short physical performance battery, and timed up-and-go [5, 19].

Diagnostic criteria for sarcopenia have been proposed by different international working groups. The International Sarcopenia Working Group defined sarcopenia as a decrease in lean tissue and physical function of the whole body or limbs (walking speed ≤ 1/s) [25].

The European Working Group for the study of Sarcopenia (EWGSOP) defined sarcopenia based on the combination of SMM, assessed using DXA or BIA, and muscle function, indicated by muscle strength or performance [24]. In the clinical setting, EWGSOP recommends the assessment of walking speed to evaluate frailty, with a threshold of < 0.8 m/s; patients with sarcopenia and impaired physical performance (gait speed ≤ 0.8 m/s) are considered to have severe sarcopenia. Regarding grip strength, the EWGSOP has proposed different cut-offs based on an individual's BMI. The 2019 updated version of the EWGSOP2 consensus recommends that muscle strength should be measured before SMM, and that sarcopenia should be suspected in patients with reduced muscle strength [29].

The Foundation for the National Institutes of Health (FNIH) Sarcopenia Project proposed to define sarcopenia as low SMM, low muscle strength, and physical decline. The FHIH recommends the use of DXA to measure SMM, with corrections for BMI [26].

The Asian Working Group for Sarcopenia (AWGS) [27] proposed diagnostic criteria for sarcopenia applicable to Asian populations, using grip strength and physical function for preliminary screening. The diagnostic process for sarcopenia proposed by the AWGS includes a detailed protocol that involves self-assessment, preliminary screening, diagnosis, and severity assessment. In the primary care setting and hospital setting, preliminary screening of sarcopenia can be based on a measurement of calf circumference (<34 cm in men, < 33 cm in women), the SARC-F scale (≥4), or SARC-Calf scale (≥11). DXA or BIA can be used to improve SMM measurements during hospitalization. The AWGS defines sarcopenia as a decrease in SMM and muscle strength or physical activity. The patients with reduced SMM and decreased muscle strength and reduced physical activity are considered to have severe sarcopenia [30].

### Diagnostic criteria for obesity

In the SO study, obesity was defined as BMI ≥ 30 kg/m^2^ [4], increased PBF (men ≥ 27% or 28%, women ≥ 35%, 38%, or 40%, depending on specific study criteria) [5, 6], and waist circumference higher than the population-specific quartile [41] or higher than the World Health Organization (WHO)-recommended waist circumference (male ≥ 102 cm, female ≥ 88 cm) [42]. The American Association of Clinical Endocrinologists (AACE) proposed the use of PBF to define obesity, where PBF > 25% and > 35% represent obesity in males and females, respectively [25]. So far, there are no cut-off points for BMI, PBF, and waist circumference for obesity in the elderly. Adipose tissue can be separated into subcutaneous adipose tissue (SAT) and visceral adipose tissue (VAT). Currently, there is a lack of relevant diagnostic guidelines to define obesity based on SAT and VAT. Indeed, some scholars have proposed that future studies should focus on distinguishing between sarcopenic subcutaneous obesity and sarcopenic visceral obesity and use the standardized VAT/SAT ratio to diagnose SO [43].

## Prevalence

Due to the heterogeneity of the definition of SO, the reported prevalence of SO is variable and ranges from 2.75% to 20% or more [19]. Further, the prevalence of SO differs according to gender, race, and age because of the different standards adopted by different countries. A systematic review reported that the global prevalence of SO in the elderly was 11% [3]. It also showed that the overall morbidity rate of SO in the elderly aged 75 and older was 23%, indicating that the prevalence of SO increases with age. The potential causes include the changes in hormones and body composition (muscle atrophy and adipose tissue accumulation) caused by aging. There was no sex difference in the prevalence of SO among the elderly, suggesting that both women and men are at a high risk [5, 6]. The prevalence of SO was higher in South America and North America, and the pooled prevalence of SO was higher in inpatients than in community residents, indicating that malnutrition and immobility are linked to the development of SO in the elderly in the hospital.

## Etiology and Pathogenesis

The etiology and pathogenesis of SO are intertwined and intricate. The core biological factors leading to SO are changes in body composition related to aging, hormonal changes, the interplay between metabolism and inflammation, environmental factors (unhealthy diet and lack of exercise), and chronic diseases [5, 6, 11, 44]. Aging and obesity cause atrophy of fast type II muscle fibers and a switch to slow type I muscle fibers and neurodegeneration, leading to loss of muscle neurotrophic effects and promotion of intramyocellular lipid (IMCL) deposition. A prominent manifestation of SO is anabolic resistance (AR), which is characterized by reduced skeletal muscle protein synthesis rates and increased muscle protein degradation rates [11, 45]. The key pathophysiological mechanism of SO is a vicious cycle between myocytes and adipocytes [6]. Obesity is characterized by the expansion of adipose tissue, which leads to adipose tissue inflammation and dysfunction, leading to the over-production of fatty acids. When the number of fatty acids exceeds the oxidation capacity of skeletal muscle, IMCL [46] is formed, and this affects the function of the GLUT4 transporter. This subsequently leads to reduced glucose utilization and increased fatty acid oxidation in mitochondria, which leads to impaired insulin sensitivity of skeletal muscle, inhibition of mitochondrial respiration, reactive oxygen species formation, muscle cytotoxicity, catabolism, and inflammation. Muscle intercellular adipose tissue and IMCL are characterized by dysregulation of adipokines and cytokines (↑TNF-α, ↑IL-6, ↑leptin, ↑IL-1β, ↑MCP-1, ↓adiponectin), which induce IR and lipotoxicity, and eventually lead to sarcopenia [47-50]. At the same time, adipose tissue enhances the secretion of pro-inflammatory actin in muscle tissue. On the other hand, myocytokines (↓IL-15, ↓irisin, ↓IGF-1, ↑myostatin, impaired IL-6 secretion) may lead to muscle atrophy and dysfunction, they may play an endocrine role to aggravate fatty tissue inflammation and propagate a pro-inflammatory state between myocytes and adipocytes [5,49-52]. A summary of the possible mechanisms is shown in Figure 1.

### Age-related changes in body composition

Under the influence of lifestyle factors and hormone levels, body composition changes significantly with age. The main changes are an increase in total fat mass, which peaks between 60 and 75 years old, and a decrease in peripheral subcutaneous fat, preferential accumulation of visceral fat, and ectopic fat infiltration in various organs. By comparison, SMM and muscle strength start to decrease from approximately 30 years of age, and the rate of decline of muscle mass accelerates significantly in adults over 60 years old [53]. Therefore, the body weight of older people is mainly composed of adipose tissue rather than lean tissue [5, 6].

### Hormonal changes

Hormonal changes related to aging include insulin resistance, decreased thyroid hormone level, and increased levels of cortisol, growth hormones, insulin-like growth factor (IGF-1), sex hormones, and dehydroisoandrosterone sulfate, which all contribute to SO. In postmenopausal women, body composition changes result in increased adipose tissue, visceral fat infiltration, and decreased SMM [5]. In men, the decline in testosterone levels with aging has an adverse effect on the distribution of muscle and adipose tissue [5].

### Inflammation and metabolism

SO is considered to represent a sub-acute, chronic pro-inflammatory state, which hinders metabolic processes (oxidative stress and insulin resistance), destroys the function of adipose and muscle, and increases the risk of chronic disease [11, 53]. Recent studies have shown that there is a key crosstalk between metabolism and inflammation, which has led to increased focus on the concept of metabolic inflammation [11]. In SO, adipocytes accumulate in muscle tissue and other organs (heart, liver and pancreas and so on) and secrete pro-inflammatory cytokines (TNF-α, IL-6, IL-1 and leptin), thus leading to the infiltration of inflammatory cells and inducing insulin resistance and lipotoxicity, which directly affects skeletal muscle and accelerates muscle protein degradation and apoptosis, and promotes muscle tissue reduction and adipose tissue accumulation through inflammation and oxidative stress [5, 6, 54, 55]. The levels of IL-6 and TNF are increased by leptin, thereby reducing the anabolism of IGF-1 [56]. The decrease in IGF-1 and age-related testosterone levels increases the incidence of frailty [57]. Adiponectin is inversely correlated with age and obesity and counteracts the effect of leptin. The increase in TNF can directly inhibit the effect of adiponectin and inhibit the synthesis of muscle proteins and mitochondrial function. Obesity can also cause leptin resistance, resulting in reduced breakdown of lipid oxidative products and ectopic fat deposition [57].

### Myocyte mechanism

Numerous molecules (TNF-α, IL-6, IL-1, adiponectin, leptin, muscle somatostatin, sex hormones (testosterone and estrogen), growth hormone, insulin and glucocorticoid, and irisin) have been implicated in the pathogenesis of SO [50]. Aging stimulates fat to infiltrate muscles, and obesity promotes fatty infiltration of other organs such as the liver, pancreas, and heart. Lipid deposition in muscle cells promotes lipotoxicity and inflammation and induces the de-differentiation of mesenchymal progenitor cells expressing adipose tissue genes. Impaired muscle regeneration capacity may lead to fibrosis of muscle tissue, impaired mitochondrial function, increased production of reactive oxygen species, upregulation of myostatin expression, impaired fatty acid oxidation, and reduced lipolysis, thereby promoting insulin resistance and impairing muscle function [5].

### Influence of environmental and chronic diseases

The onset of SO is influenced by several lifestyle factors, of which the most important are dietary changes and lack of physical activity. Aging itself leads to obesity and reduced physical activity. Further, the dietary pattern of elderly people, which is often characterized by insufficient protein intake combined with excess dietary calorie intake that is rich in saturated fatty acids, coupled with a sedentary lifestyle promotes the occurrence of sarcopenic obesity [11]. SO shares a common pathogenic mechanism with a variety of chronic diseases such as diabetes, cardiovascular disease, and cancer, among others [7, 8, 49]. SO can lead to a variety of pathophysiological changes, such as excessive secretion of pro-inflammatory cytokines by adipose tissue, changes in the expression of adipocytokines by adipocytes, and fat accumulation in muscle [6, 12]. Skeletal muscle cell atrophy reduces the expression of GLUT4 in muscle tissue and decreases the demand for insulin-dependent glucose uptake [58]. The pro-inflammatory state and lipid accumulation in muscle fibers induce phosphorylation and deactivate insulin receptors and their substrates [19], resulting in insulin resistance and AR. Insulin resistance is the core mechanism of SO associated with cardiovascular metabolic diseases and cancer [49, 50].

## Preventive and therapeutic strategies

At present, the optimal treatment of SO has not been established. Nutritional interventions, such as a hypocaloric diet, and exercise training or physical therapy are the mainstay of SO prevention and treatment to achieve changes in body composition (muscle gain and fat reduction) and improve the functional status and quality of life of elderly patients. However, solely focusing on weight loss per se is not desirable for the elderly because weight loss may actually pose health risks such as loss of muscle and bone mass.

### Diet intervention strategies

The nutritional strategies for the prevention of SO in the elderly include hypocaloric diets and high protein and micronutrients supplementation [5, 6]. Extremely low-calorie diets and rapid calorie restriction for the management of sarcopenic obese older adults are strongly discouraged, because they can have harmful effects on SMM, bone mineral density, and the micronutrient status, and increase the risk of hypovolemia and electrolyte disorders. [5, 6]. Instead, the optimal and safe range of calorie restriction is about 200-750 kcal per day [59]. It is recommended that elderly people should consume larger amounts of high-quality protein (aiming for 1-1.2 g/kg/d) [5], with an even higher intake (1.2-1.5 g/kg/d) [60] recommended for elderly patients with sarcopenia or other chronic diseases; however, patients with renal insufficiency should monitor their protein intake. Intake of dietary essential amino acids (EAAs), and especially high leucine content, promotes muscle protein synthesis [5]. Ensuring sufficient intake of trace elements could improve several sarcopenic parameters and physical frailty, with most guidelines recommending supplementation with 1200 mg of calcium and vitamin D (800 to 1000 IU daily) [61]. The American Academy of Geriatrics recommends a daily intake of vitamin D3 (1,000 IU) and calcium in the elderly non-hospitalized population older than 65 years to maintain serum vitamin D levels ≥ 30 ng/ml [62]. A recent review proposed a new approach to dietary recommendations based on the gut microbiota profile in patients with SO based on the finding that a high-protein diet with an elevated concentration of EAA and increased dietary fiber intake may promote the eugenics of the intestinal microbiota [63].

### Exercise interventions

Physical activity (aerobic exercise, resistance exercise, and combination training) is a powerful treatment strategy to counteract one or more of the biological effects of SO and has been demonstrated to promote insulin sensitivity [7, 64], reduce oxidative stress [65], induce mitochondrial biosynthesis, ameliorate inflammation, and eliminate muscle cell apoptosis, among other positive effects [5, 58, 63-66].

Aerobic exercise can ameliorate cardiopulmonary function and reduce mortality [5, 67], and resistance training is effective in enhancing muscle function and strength in the elderly [68]. Because the elderly often suffer from various chronic diseases, a tailored exercise program that considers these comorbidities and associated physical limitations is recommended by most guidelines. Aerobic exercise should aim for a peak heart rate of 65% with a target heart rate zone of 70%-85% of the peak. On the other hand, resistance training should focus on 1-2 muscle groups and include 8-12 repetitions, with an initial intensity of 65% of 1 repetition maximum (1RM), aiming to reach 2-3 repetitions with an intensity of 75% 1RM. Resistance training should aim to achieve fatigue rather than exhaustion to prevent musculoskeletal injury [60, 69].

### Emerging treatments for SO

Although many emerging pharmacological interventions have been studied, such as testosterone supplementation [13], selective androgen receptor modulators [14], myostatin inhibitors [15], and anti-obesity drugs [5, 6], there are no approved drugs for the treatment of SO in the elderly. A recent systematic review of treatment strategies for SO showed that electrical acupuncture and whole-body electromyostimulation associated with nutritional supplementation are new and effective strategies to induce changes in body composition [16].

Whole body vibration therapy has become a safe and convenient technique to cause neuromuscular activation and simulate the contraction of skeletal muscle [70]. A randomized controlled clinical trial of 90 elderly men found that whole-body vibration therapy significantly increased muscle strength and physical function in older people [71]. This therapy has the potential to replace traditional exercise for the treatment of SO among elderly people as it is better tolerated and can reduce fat mass (FM) and increase muscle strength. Nevertheless, this therapy is still in the research stage and there is a need for further clinical studies to verify its efficacy in routine clinical practice.

A recent study demonstrated that the adenosine A2B receptor (A2B) is highly expressed in muscle tissue and brown adipose tissue (BAT) and may be a target for SO [17]. Adenosine / A2B signaling pathway display the core function in maintaining the quality and function of skeletal muscle. Stimulation of A2B could play a key role in anti-aging and anti-obesity effects and restore skeletal muscle function and quality to adolescent levels. At the same time, A2B activation also reduced the impaired BAT function and induced white adipose tissue browning associated with age and obesity. However, the current evidence regarding the role of adenosine/A2B signaling is limited to animal studies, and more research is needed to verify the role of this signaling pathway in humans.

## Conclusions and perspectives

The prevalence of SO increases with age and it is estimated that more than one-tenth of the elderly population suffer from SO globally. This has important public health consequences as SO is associated with frailty, falls, disability, and increased morbidity and mortality, and places a heavy burden on individuals, society, and the medical system. To further our understanding of SO, it is essential that clinicians and researchers establish a universal consensus for the definition and diagnosis of SO and focus on SO screening to identify susceptible individuals early. Additional studies are warranted to clarify the pathogenesis of SO and formulate the best diet and exercise intervention scheme to provide tailored treatments and promote healthy aging. In conclusion, establishing an accurate definition and diagnostic criteria for SO, and introducing effective preventative and treatment options have become an urgent task for researchers and clinicians.

## References

[1] United Nations Department of Economic and Social Affairs (DESA)/Population Division (2019). World population prospects. https://population.un.org/wpp/Download/Standard/Population. 2019.

[2] Kaeberlein M, Rabinovitch PS, Martin GM (2015). Healthy aging: The ultimate preventative medicine. Science, 350:1191-1193. 26785476 10.1126/science.aad3267 PMC4793924

[3] Gao Q, Mei F, Shang Y, et al. (2021). Global prevalence of sarcopenic obesity in older adults: A systematic review and meta-analysis. Clin Nutr, 40:4633-4641. 34229269 10.1016/j.clnu.2021.06.009

[4] Zamboni M, Mazzali G, Fantin F, et al. (2008) Sarcopenic obesity: A new category of obesity in the elderly. Nutr Metab Cardiovasc Dis, 18:388-395. 18395429 10.1016/j.numecd.2007.10.002

[5] Batsis JA, Villareal DT (2018). Sarcopenic obesity in older adults: aetiology, epidemiology and treatment strategies. Nat Rev Endocrinol, 14:513-537. 30065268 10.1038/s41574-018-0062-9 PMC6241236

[6] Koliaki C, Liatis S, Dalamaga M, et al. (2019). Sarcopenic Obesity: Epidemiologic Evidence, Pathophysiology, and Therapeutic Perspectives.Curr Obes Rep, 8:458-471. 31654335 10.1007/s13679-019-00359-9

[7] Wang M, Tan Y, Shi Y, et al. (2020). Diabetes and Sarcopenic Obesity: Pathogenesis, Diagnosis, and Treatments. Front Endocrinol (Lausanne), 11:568. 32982969 10.3389/fendo.2020.00568 PMC7477770

[8] Atkins JL, Wannamathee SG (2020). Sarcopenic obesity in ageing: cardiovascular outcomes and mortality. Br J Nutr, 124:1102-1113. 32616084 10.1017/S0007114520002172

[9] Petermann-Rocha F, Yang S, Gray S R, et al. (2021). Sarcopenic obesity and its association with respiratory disease incidence and mortality - Authors' reply. Clin Nutr, 40:2520. 33932797 10.1016/j.clnu.2021.03.030

[10] Guo A, Li K, Xiao Q (2020). Sarcopenic obesity: Myokines as potential diagnostic biomarkers and therapeutic targets? Exp Gerontol, 139:111022. 32707318 10.1016/j.exger.2020.111022

[11] Lynch G M, Murphy C H, Castro E D M, et al. (2020). Inflammation and metabolism: the role of adiposity in sarcopenic obesity. Proc Nutr Soc, 79:435-447. 10.1017/S0029665120007119 32669148

[12] Kalinkovich A, Livshits G (2017). Sarcopenic obesity or obese sarcopenia: A cross talk between age-associated adipose tissue and skeletal muscle inflammation as a main mechanism of the pathogenesis. Ageing Res Rev, 35:200-221. 27702700 10.1016/j.arr.2016.09.008

[13] Neto WK, Gama EF, Rocha LY, et al. (2015). Effects of testosterone on lean mass gain in elderly men: systematic review with meta-analysis of controlled and randomized studies. AGE, 37:9742 25637335 10.1007/s11357-014-9742-0 PMC4312307

[14] PapanicolaouDA, AtherSN, ZhuH, et al. (2013). A phase IIA randomized, placebo-controlled clinical trial to study the efficacy and safety of the selective androgen receptor modulator (SARM), MK-0773 in female participants with sarcopenia. J Nutr Health Aging, 17:533-543.2373255010.1007/s12603-013-0335-x

[15] Camporez JG, Petersen MC, Abudukadier A, et al. (2016). Anti-myostatin antibody increases muscle mass and strength and improves insulin sensitivity in old mice. PNAS, 113:2212-2217. 26858428 10.1073/pnas.1525795113 PMC4776508

[16] Poggiogalle E, Parrinello E, Barazzoni R, et al. (2021). Therapeutic strategies for sarcopenic obesity: a systematic review. Curr Opin Clin Nutr Metab Care, 24:33-41. 33323715 10.1097/MCO.0000000000000714

[17] Gnad T, Navarro G, Lahesmaa M, et al. (2020). Adenosine/A2B Receptor Signaling Ameliorates the Effects of Aging and Counteracts Obesity. Cell Metab, 32:56-70. 32589947 10.1016/j.cmet.2020.06.006 PMC7437516

[18] Baumgartnera RN (2000). Body Composition in Healthy Aging. Ann N Y Acad Sci, 904:437-448. 10865787 10.1111/j.1749-6632.2000.tb06498.x

[19] Donini L M, Busetto L, Bauer J M, et al. (2020). Critical appraisal of definitions and diagnostic criteria for sarcopenic obesity based on a systematic review. Clin Nutr, 39:2368-2388. 31813698 10.1016/j.clnu.2019.11.024

[20] Waters DL, Baumgartner RN (2011). Sarcopenia and Obesity.Clin Geriatr Med, 27:401-421. 21824555 10.1016/j.cger.2011.03.007

[21] Newman A B, Kupelian V, Visser M, et al. (2003). Sarcopenia: alternative definitions and associations with lower extremity function. J Am Geriatr Soc, 51:1602-1609. 14687390 10.1046/j.1532-5415.2003.51534.x

[22] Baumgartner RN, Wayne SJ, Waters DL, et al. (2004). Sarcopenic obesity predicts instrumental activities of daily living disability in the elderly. Obes Res, 12:1995-2004. 15687401 10.1038/oby.2004.250

[23] Kim TN, Yang SJ, Yoo HJ, et al. (2009). Prevalence of sarcopenia and sarcopenic obesity in Korean adults: the Korean sarcopenic obesity study. Int J Obes (Lond), 33:885-892. 19564878 10.1038/ijo.2009.130

[24] Cruz-Jentoft AJ, Baeyens JP, Bauer JM, et al. (2010). Sarcopenia: European consensus on definition and diagnosis: Report of the European Working Group on Sarcopenia in Older People. Age Ageing, 39:412-423. 20392703 10.1093/ageing/afq034 PMC2886201

[25] Fielding RA, Vellas B, Evans WJ, et al. (2011). Sarcopenia: An Undiagnosed Condition in Older Adults. Current Consensus Definition: Prevalence, Etiology, and Consequences. International Working Group on Sarcopenia. J Am Med Dir Assoc, 12:249-256. 21527165 10.1016/j.jamda.2011.01.003 PMC3377163

[26] Studenski SA, Peters K W, Alley DE, et al. (2014). The FNIH Sarcopenia Project: Rationale, Study Description, Conference Recommendations, and Final Estimates. J Gerontol A Biol Sci Med Sci, 69:547-558. 24737557 10.1093/gerona/glu010 PMC3991146

[27] Chen L, Liu L, Woo J, et al. (2014). Sarcopenia in Asia: Consensus Report of the Asian Working Group for Sarcopenia. J Am Med Dir Assoc,15:95-101. 24461239 10.1016/j.jamda.2013.11.025

[28] Chuang SY, Hsu YY, Chen RC, et al. (2016). Abdominal Obesity and Low Skeletal Muscle Mass Jointly Predict Total Mortality and Cardiovascular Mortality in an Elderly Asian Population. J Gerontol A Biol Sci Med Sci, 71:1049 26590913 10.1093/gerona/glv192

[29] Cruz-Jentoft A J, Bahat G, Bauer J, et al. (2019). Sarcopenia: revised European consensus on definition and diagnosis. Age Ageing, 48:16-31. 30312372 10.1093/ageing/afy169 PMC6322506

[30] Chen LK, Woo J, Assantachai P, et al. (2020). Asian Working Group for Sarcopenia: 2019 Consensus Update on Sarcopenia Diagnosis and Treatment. J Am Med Dir Assoc, 21:300-307. 32033882 10.1016/j.jamda.2019.12.012

[31] Kendler DL, Borges JLC, Fielding RA, et al. (2013). The Official Positions of the International Society for Clinical Densitometry: Indications of Use and Reporting of DXA for Body Composition. J Clin Densitom, 16:496-507. 24090645 10.1016/j.jocd.2013.08.020

[32] Bosy-Westphal A, Jensen B, Braun W, et al. (2017). Quantification of whole-body and segmental skeletal muscle mass using phase-sensitive 8-electrode medical bioelectrical impedance devices. Eur J Clin Nutr, 71:1061-1067. 28327564 10.1038/ejcn.2017.27 PMC5589975

[33] Woodrow G (2009). Body composition analysis techniques in the aged adult: indications and limitations. Curr Opin Clin Nutr Metab Care, 12:8-14. 19057181 10.1097/MCO.0b013e32831b9c5b

[34] Rosenberg IH (1989). Summary comments: epidemiological and methodological problems in determining nutritional status of older persons. Am J Clin Nutr, 50:1231-1233.

[35] Cruz-Jentoft AJ, Sayer AA (2019). Sarcopenia. Lancet, 393:2636-2646. 31171417 10.1016/S0140-6736(19)31138-9

[36] Vellas B, Fielding RA, Bens C, et al. (2018). Implications of ICD-10 for Sarcopenia Clinical Practice and Clinical Trials: Report by the International Conference on Frailty and Sarcopenia Research Task Force. J Frailty Aging, 7:2. 29412436 10.14283/jfa.2017.30

[37] Ryu M, Jo J, Lee Y, et al. (2013). Association of physical activity with sarcopenia and sarcopenic obesity in community-dwelling older adults: the Fourth Korea National Health and Nutrition Examination Survey. Age Ageing, 42:734-740. 23761456 10.1093/ageing/aft063

[38] Delmonico M J, Harris T B, Lee J, et al. (2007). Alternative Definitions of Sarcopenia, Lower Extremity Performance, and Functional Impairment with Aging in Older Men and Women. J Am Geriatr Soc, 55:769-774. 17493199 10.1111/j.1532-5415.2007.01140.x

[39] Newman AB, Kupelian V, Visser M, et al. (2006). Strength, but not muscle mass, is associated with mortality in the health, aging and body composition study cohort. The journals of gerontology. Series A, J Gerontol A Biol Sci Med Sci, 61:72-77. 16456196 10.1093/gerona/61.1.72

[40] Batsis JA, Zbehlik AJ, Pidgeon D, et al. (2015). Dynapenic obesity and the effect on long-term physical function and quality of life: data from the osteoarthritis initiative. BMC Geriatr, 15:118. 26449277 10.1186/s12877-015-0118-9 PMC4599326

[41] Schrager MA, Metter EJ, Simonsick E, et al. (2007). Sarcopenic obesity and inflammation in the InCHIANTI study. J Appl Physiol, 102:919-925. 17095641 10.1152/japplphysiol.00627.2006 PMC2645665

[42] WHO (2000). Obesity: preventing and managing the global epidemic. World Health Organ Tech Rep Ser, 15:18-30. 11234459

[43] Perna S, Spadaccini D, Rondanelli M (2019). Sarcopenic obesity: time to target the phenotypes. J Cachexia Sarcopenia Muscle, 10:710-711. 30941929 10.1002/jcsm.12425 PMC6596395

[44] Guo A, Li K, Xiao Q (2020). Sarcopenic obesity: Myokines as potential diagnostic biomarkers and therapeutic targets? Exp Gerontol, 139:111022. 32707318 10.1016/j.exger.2020.111022

[45] Murton AJ, Marimuthu K, Mallinson JE, et al. (2015). Obesity Appears to Be Associated With Altered Muscle Protein Synthetic and Breakdown Responses to Increased Nutrient Delivery in Older Men, but Not Reduced Muscle Mass or Contractile Function. Diabetes, 64:3160-3171. 26015550 10.2337/db15-0021

[46] Gemmink A, Goodpaster BH, Schrauwen P, Hesselink MKC (2017). Intramyocellular lipid droplets and insulin sensitivity, the human perspective. Biochim Biophys Acta Mol Cell Biol Lipids. 1862:1242-1249. 28739280 10.1016/j.bbalip.2017.07.010

[47] Rivas DA, McDonald DJ, Rice NP, Haran PH, Dolnikowski GG, Fielding RA (2016). Diminished anabolic signaling response to insulin induced by intramuscular lipid accumulation is associated with inflammation in aging but not obesity. Am J Physiol Regul Integr Comp Physiol, 310:R561-R569. 26764052 10.1152/ajpregu.00198.2015 PMC4867383

[48] Yamauchi T, Kamon J, Minokoshi Y, Ito Y, Waki H, Uchida S, et al. (2002). Adiponectin stimulates glucose utilization and fatty-acid oxidation by activating AMP-activated protein kinase. Nat Med, 8:1288-1295. 12368907 10.1038/nm788

[49] Silveira EA, Da SFR, Spexoto M, et al. (2021). The Role of Sarcopenic Obesity in Cancer and Cardiovascular Disease: A Synthesis of the Evidence on Pathophysiological Aspects and Clinical Implications. Int J Mol Sci, 22(9). 10.3390/ijms22094339 PMC8122649 33919368

[50] Hong SH, Choi KM (2020). Sarcopenic Obesity, Insulin Resistance, and Their Implications in Cardiovascular and Metabolic Consequences. Int J Mol Sci, 21(2). 10.3390/ijms21020494 PMC7013734 31941015

[51] Kurdiova T, Balaz M, Vician M, Maderova D, Vlcek M, Valkovic L, et al. (2014). Effects of obesity, diabetes and exercise on Fndc5 gene expression and irisin release in human skeletal muscle and adipose tissue: In vivo and in vitro studies. [J] Physiol. 592:1091-1111 10.1113/jphysiol.2013.264655 PMC3948565 24297848

[52] Kraakman MJ, Kammoun HL, Allen TL, Deswaerte V, Henstridge DC, Estevez E, et al. (2015). Blocking IL-6 trans-signaling prevents high-fat diet-induced adipose tissue macrophage recruitment but does not improve insulin resistance. Cell Metab. 21:403-416.07. 25738456 10.1016/j.cmet.2015.02.006

[53] Xie WQ, Xiao GL, Fan Y B, et al. (2021). Sarcopenic obesity: research advances in pathogenesis and diagnostic criteria. Aging Clin Exp Res, 33:247-252. 31845200 10.1007/s40520-019-01435-9

[54] Barazzoni R, Bischoff S, Boirie Y, et al. (2018). Sarcopenic Obesity: Time to Meet the Challenge. Obesity Facts, 11:294-305. 30016792 10.1159/000490361 PMC6189532

[55] Sakuma K, Yamaguchi A (2013). Sarcopenic Obesity and Endocrinal Adaptation with Age. Int J Endocrinol, 2013:1-12. 10.1155/2013/204164 PMC3639625 23690769

[56] Hamrick MW (2017). Role of the Cytokine-like Hormone Leptin in Muscle-bone Crosstalk with Aging. J Bone Metab, 24:1. 28326295 10.11005/jbm.2017.24.1.1 PMC5357607

[57] Yeap BB, Paul Chubb SA, Lopez D, et al. (2013). Associations of insulin-like growth factor-I and its binding proteins and testosterone with frailty in older men. Clin Endocrinol (Oxf), 78:752-759. 23009592 10.1111/cen.12052

[58] Thornell L (2011). Sarcopenic obesity: satellite cells in the aging muscle. Curr Opin Clin Nutr Metab Care, 14:22-27. 21088571 10.1097/MCO.0b013e3283412260

[59] Freiberger E, Goisser S, Porzel S, et al. (2015). Sarcopenic obesity and complex interventions with nutrition and exercise in community-dwelling older persons &ndash; a narrative review. Clin Interv Aging, 2015:1267. 10.2147/CIA.S82454 PMC4531044 26346071

[60] Trouwborst I, Verreijen A, Memelink R, et al. (2018). Exercise and Nutrition Strategies to Counteract Sarcopenic Obesity. Nutrients, 10(5). 10.3390/nu10050605 PMC5986485 29757230

[61] Reidenberg MM (2013). Vitamin D and Calcium Supplementation to Prevent Fractures in Adults. Ann Intern Med, 159(12). 10.7326/0003-4819-159-12-201312170-00015 24343397

[62] American Geriatrics Society Workgroup on Vitamin D Supplementation for Older Adults (2014). Recommendations Abstracted from the American Geriatrics Society Consensus Statement on Vitamin D for Prevention of Falls and Their Consequences. J Am Geriatr Soc, 62:147-152. 24350602 10.1111/jgs.12631

[63] Prokopidis K, Cervo MM, Gandham A, et al. (2020). Impact of Protein Intake in Older Adults with Sarcopenia and Obesity: A Gut Microbiota Perspective. Nutrients, 12(8). 10.3390/nu12082285 PMC7468805 32751533

[64] Poggiogalle E, Parrinello E, Barazzoni R, et al. (2021). Therapeutic strategies for sarcopenic obesity: a systematic review. Curr Opin Clin Nutr Metab Care, 24:33-41. 33323715 10.1097/MCO.0000000000000714

[65] Lee D, Shook RP, Drenowatz C, et al. (2016). Physical activity and sarcopenic obesity: definition, assessment, prevalence and mechanism. Future Sci OA, 2:O127. 10.4155/fsoa-2016-0028 PMC5137918 28031974

[66] Joseph A, Adhihetty PJ, Leeuwenburgh C (2016). Beneficial effects of exercise on age-related mitochondrial dysfunction and oxidative stress in skeletal muscle. J Physiol, 594:5105-5123. 26503074 10.1113/JP270659 PMC5023701

[67] Chen H, Chung Y, Chen Y, et al. (2017). Effects of Different Types of Exercise on Body Composition, Muscle Strength, and IGF-1 in the Elderly with Sarcopenic Obesity. J Am Geriatr Soc, 65:827-832. 28205203 10.1111/jgs.14722

[68] Hsu K, Liao C, Tsai M, et al. (2019). Effects of Exercise and Nutritional Intervention on Body Composition, Metabolic Health, and Physical Performance in Adults with Sarcopenic Obesity: A Meta-Analysis. Nutrients, 11:2163. 10.3390/nu11092163 PMC6770949 31505890

[69] Villareal DT, Aguirre L, Gurney AB, et al. (2017). Aerobic or Resistance Exercise, or Both, in Dieting Obese Older Adults. N Engl J Med, 376:1943-1955. 28514618 10.1056/NEJMoa1616338 PMC5552187

[70] Verschueren S M, Bogaerts A, Delecluse C, et al. (2011). The effects of whole-body vibration training and vitamin D supplementation on muscle strength, muscle mass, and bone density in institutionalized elderly women: A 6-month randomized, controlled trial. J Bone Miner Res, 26:42-49. 20648661 10.1002/jbmr.181

[71] Zhu Y, Peng N, Zhou M, et al. (2019). Tai Chi and whole-body vibrating therapy in sarcopenic men in advanced old age: a clinical randomized controlled trial. Eur J Ageing, 16:273-282. 31543722 10.1007/s10433-019-00498-x PMC6728405

